# The Effects of Stimulator, Waveform, and Current Direction on Intracortical Inhibition and Facilitation: A TMS Comparison Study

**DOI:** 10.3389/fnins.2019.00703

**Published:** 2019-07-09

**Authors:** Maximilian J. Wessel, Laurijn R. Draaisma, Takuya Morishita, Friedhelm C. Hummel

**Affiliations:** ^1^Defitech Chair of Clinical Neuroengineering, Center for Neuroprosthetics and Brain Mind Institute, Swiss Federal Institute of Technology (EPFL), Geneva, Switzerland; ^2^Defitech Chair of Clinical Neuroengineering, Clinique Romande de Réadaptation, Center for Neuroprosthetics and Brain Mind Institute, Swiss Federal Institute of Technology (EPFL Valais), Sion, Switzerland; ^3^Department of Clinical Neuroscience, University of Geneva Medical School, Geneva, Switzerland

**Keywords:** transcranial magnetic stimulation, short intracortical inhibition, intracortical facilitation, Magstim, MagVenture, comparison

## Abstract

**Background:** Cortical function is dependent on the balance between excitatory and inhibitory influences. In the human motor cortex, surrogates of these interactions can be measured *in vivo*, non-invasively with double-pulse transcranial magnetic stimulation (TMS). To compare results from data acquired with different available setups and bring data together, it is inevitable to determine whether different TMS setups lead to comparable or differential results.

**Objective:** We assessed and compared short intracortical inhibition (SICI) and intracortical facilitation (ICF) testing four different experimental conditions.

**Methods:** SICI and ICF were studied with different stimulators (Magstim BiStim^2^ or MagVenture MagPro X100), waveforms (monophasic or biphasic), current directions (anterior-posterior or posterior-anterior) at interstimulus intervals (ISIs) of 1, 3, 10, 15 ms.

**Results:** We were not able to detect differences for SICI and ICF, when comparing the tested conditions, except for 3 ms SICI in which the anterior-posterior current direction led to stronger modulation. Correlation analysis suggested comparability for 3 ms SICI for the Magstim monophasic posterior-anterior condition with both tested MagVenture conditions.

**Conclusions:** 3 ms SICI data sets obtained with two different, commonly used stimulators (Magstim BiStim^2^ or MagVenture MagPro X100) with conventionally used stimulation parameters are largely comparable. This may allow the combination of data sets in an open science view.

## Introduction

Inhibitory and excitatory interactions are key components of cortical processing (Kirkwood, 2015). With the use of double-pulse transcranial magnetic stimulation (dpTMS), it is possible to assess correlates of these interactions within the human motor cortex *in vivo* and non-invasively, first described by Kujirai et al. (1993), Chen (2004). It pairs a subthreshold conditioning stimulus (CS) with a subsequent suprathreshold test stimulus (TS). The test response, mainly measured via the amplitude of motor evoked potentials (MEPs), is inhibited at shorter interstimulus intervals (ISIs) of 1–6 ms, this effect is commonly termed short intracortical inhibition (SICI). At longer ISIs of 8–30 ms the test response is facilitated, here referred to as intracortical facilitation (ICF). SICI and ICF have been widely used to study motor cortex physiology in healthy subjects and neurological disorders, e.g., (Hummel et al., 2009; Heise et al., 2010, 2013). SICI has been associated with GABA_A_ and ICF with glutamatergic neurotransmission (Ziemann et al., 1998; Di Lazzaro et al., 2000). Importantly, this neurotransmission has been linked to various aspects of human behavior, such as regulation of learning, memory, cognition, or emotions (Ende, 2015).

SICI and ICF are frequently used to study neurological conditions and to validate neurotechnological interventions, such as transcranial direct current stimulation, e.g., (Zimerman et al., 2012). However, as different devices and protocol parameters are used, it is important to determine whether these different devices and protocols lead to comparable data. This would be a crucial prerequisite to judge whether results derived from different experimental setups are comparable. Furthermore, this knowledge will pave the way to comprehensively combine data sets from different sources, e.g., toward open science approaches. Even more importantly, it is a necessary basis for the potential of SICI and ICF as diagnostic tools or biomarkers to predict recovery and treatment response.

Comparative studies of different devices have been conducted to assess motor thresholds, MEP amplitudes, MEP latencies, or TMS-evoked potentials (Kammer et al., 2001; Van Doren et al., 2015). For example, Van Doren et al. (2015) reported a higher magnetic field strength, a shorter magnetic flux duration, lower motor threshold, shorter recovery time from the TMS artifact, a shorter MEP latency, and a reversed first artifact trajectory comparing the MagVenture MagPro with two other devices (the Magstim Rapid and the Deymed DuoMag XT-100 stimulator) operating in biphasic mode.

To the best of our knowledge, however, the effects of the parameter interactions between stimulator, waveform, and current direction have not been studied yet for SICI and ICF. Therefore, our objective was to compliment the available literature by investigating the effects of stimulator, waveform, induced current direction, and ISI on SICI and ICF. We compared two commonly used TMS stimulators, the Magstim BiStim^2^ stimulator (Whitland, United Kingdom) and the MagVenture MagPro X100 stimulator (Farum, Denmark). In addition to the commonly used monophasic waveform, we tested a biphasic waveform, which may provide the benefit of lower values for motor thresholds (Sommer et al., 2006). We assessed the effects of the induced current direction, since on the contrary to the most commonly applied posterior to anterior (PA) currents, single-pulse anterior to posterior (AP) currents and SICI rather influence later I-waves (Nakamura et al., 1997; Sakai et al., 1997). Furthermore, we assessed different ISIs to conclude on phase-specific effects.

The aim of the present study was to make inferences about setup related confounds and emphasize between center and study comparability, when assessing SICI or ICF in future.

## Materials and Methods

### Participants

Fifteen young, healthy, right-handed participants were recruited for the study [eight female, mean age 25.20 years, mean laterality quotient Edinburgh handedness inventory 87.63 (Oldfield, 1971)]. The inclusion criteria were as follows: ≥18 and <35 years, right-handedness, normal values of Mini-mental state examination (>26/30), absence of contraindication for transcranial electric stimulation (tES), transcranial magnetic stimulation (TMS) or magnetic resonance imaging. The exclusion criteria were: presence of neuropsychiatric diseases, history of seizures, intake medication that potentially interacts with tES or TMS, musculoskeletal dysfunction that compromise finger movement, pregnancy, professional musician or intense professional usage of a computer keyboard, intake of narcotic drugs, request of not being informed in case of incidental findings. All subjects gave written informed consent in accordance with the Declaration of Helsinki (World Medical Association, 2013). The protocol was approved by the cantonal ethics committee Vaud, Switzerland (project number 2017-00765).

### Experimental Design

**FIGURE 1:**
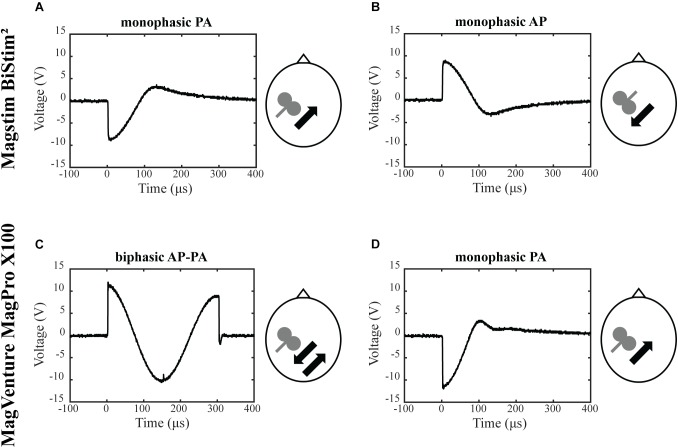
Experimental setup. Depicted are the four experimental conditions. **(A)** Magstim BiStim^2^ stimulator, monophasic waveform, posterior-anterior current direction. **(B)** Magstim BiStim^2^ stimulator, monophasic waveform, anterior-posterior current direction. **(C)** MagVenture MagPro X100 stimulator, biphasic waveform, anterior-posterior to posterior-anterior current direction. **(D)** MagVenture MagPro X100 stimulator, monophasic waveform, posterior-anterior current direction. The waveforms were measured with a probe fixed above at the coil wire intersection and a single TMS pulse applied at 50% of MSO. Arrows indicate the current directions as induced in the underlying brain tissue.

The objective was to assess the effects of different TMS conditions on SICI and ICF. The following conditions were tested and compared: (A) Magstim BiStim^2^ stimulator (Whitland, United Kingdom) with a monophasic waveform and a posterior-anterior current direction (MS PA). (B) Magstim BiStim^2^ stimulator (Whitland, United Kingdom) with a monophasic waveform and an anterior-posterior current direction (MS AP). (C) MagVenture MagPro X100 stimulator (Farum, Denmark) with a biphasic waveform and an anterior-posterior to posterior-anterior current direction (MV AP-PA). (D) MagVenture MagPro X100 stimulator (Farum, Denmark) with a monophasic waveform and a posterior-anterior current direction (MV PA), please see also Figure 1. The current direction is indicated as induced in the underlying brain tissue throughout the manuscript. The assessments were grouped into one session per stimulator. The order of the respective configurations and sessions followed a pseudorandomized sequence.

### Transcranial Magnetic Stimulation

A double-pulse protocol was utilized to assess SICI and ICF at rest (Kujirai et al., 1993). In this protocol, a subthreshold CS was followed by a suprathreshold TS. SICI was tested at ISIs of 1 and 3 ms. ICF at ISIs of 10 and 15 ms, except for MV PA in which technical limitations of the stimulator restrained us from testing a 1 ms interval. The CS was adjusted to 80% of resting motor threshold (RMT) (Kujirai et al., 1993). The RMT was defined as the minimal output of the stimulator that elicited MEPs with peak-to-peak amplitude of ≥50 μV in at least 5 out of 10 consecutive trials (Groppa et al., 2012). The TS was adjusted to evoke MEPs of ∼1 mV (Sanger et al., 2001). The TMS pulses were applied over the left motor hotspot with a figure-of-eight coil. For the MagVenture setup, we used a MC-B70 Butterfly Coil and for the Magstim setup a D70 Alpha Flat Coil, for a comparison of coil specifications please see Table 1. The coil was oriented that the handle pointed backward and ∼45° to the midsagittal line. Twenty trials were recorded for the TS and for each double-pulse paradigm with at an inter-trial-interval of 7 s ± 25%. The order followed a pseudorandom sequence, except for the first two participants in which we used a block randomization for MV PA, due to an earlier version of our trigger setup.

**Table 1:** Technical specifications of utilized TMS coils.

Manufacturer	Coil type	Averaged inductance	Focality (half-value spread S_1/2_) (Deng et al., 2013)	Stimulation depth (half-value depth, d_1/2_) (Deng et al., 2013)	Coil wing external diameter	Angle	Wire loops overlap (Thielscher and Kammer, 2004)
Magstim	D70 Alpha Flat Coil (uncoated)	16 μH	14.8 cm^2^	1.41 cm	90 mm	180°	No
MagVenture	MC-B70 Butterfly Coil	11.9 μH	13.9 cm^2^	1.35 cm	97 mm	150°	Yes

*The inductance values were provided by the respective manufacturer. Most prominent difference is the slight bend of the surface of the MC-B70 Butterfly Coil and the overlapping wire loops, which leads to a slight increase in focality.*

### EMG Recording

MEPs were recorded from the right first dorsal interosseous (FDI) muscles via surface electrodes positioned in belly tendon montage. The signal was recorded with a Noraxon DTS Receiver (Scottsdale, AZ, United States) (gain 500, sampling rate 3000 Hz, high-pass filter: 10 Hz analog Sallen-Key, low-pass filter: 1000 Hz digital FIR 128th order Butterworth) and for further processing transferred and saved on a laptop via CED Signal software (version 6.05a, Cambridge, United Kingdom).

### TMS Pulse Characterization

The applied TMS waveforms were characterized using a MagVenture MagProbe 3D (Farum, Denmark), with the probe fixed on the intersection of the respective figure-of-eight coil. For the recording of the pulse shapes, the stimulator output was set to 50% of maximum stimulator output (MSO).

### Normalization of RMT Between Stimulators

In order to compare the stimulator intensities used for the RMT and to reach a 1 mV TS, the values were normalized by the square root of the maximum energy (W) stored in capacitor (Kammer et al., 2001). For Magstim *W* = 578.1 joules and for MagVenture *W* = 300 joules, as provided by the respective manufacturer.

### Data Processing

The data were analyzed offline. The EMG time series were exported to MATLAB (version 2018a, Natick, MA, United States) and analyzed using a custom-designed graphical user interface. All trials were visually inspected. Trials with muscle pre-activation exceeding ± 25 μV from baseline <100 ms and/or ± 100 μV from baseline 500–100 ms before the TMS pulse (Hallett, 2007), trials with technical artifacts, no clear MEPs for the TS and ICF conditions [in analogy to Rossini criterion: peak-to-peak amplitude <50 μV, for review please see (Groppa et al., 2012)], or with documented suboptimal coil placement were rejected from further analysis. The MEP peak-to-peak amplitude was computed in a response window of TMS pulse +20 ms to +50 ms. The resulting peak-to-peak amplitude was averaged per condition. To indicate inhibitory or excitatory modulation, the SICI and ICF conditions were contrasted to the average TS MEPs amplitude and expressed as mean (CS+TS) / mean (TS) ^∗^ 100.

### Statistical Analysis

The statistical analysis was performed with the R software environment (version 3.5.1., 2018) (R Core Team, 2013), correlations and quality control was done with the statistical software JASP (JASP Team, 2018). Statistical significance was assumed at *p* < 0.05. The normality of the distributions was checked with the Shapiro–Wilk test. Normally distributed data were analyzed with a repeated measures analysis of variance (RM-ANOVA), applying pairwise *t*-test *post hoc* comparisons, bonferroni-corrected. Non-normally distributed data were analyzed with a Friedman test, with Wilcoxon–signed rank *post hoc* tests, bonferroni-corrected. To further assess for potential associations between conditions, we calculated Spearman’s rank-order correlations, bonferroni-corrected. Conditions that showed significant difference in the main analysis were not taken into account for the correlations. For all the analyses that involved the MS AP condition the data of one participant has been not considered for further statistical processing due to missing values (high motor threshold). Differences between conditions were tested for every ISI separately. Conditions were interpreted as comparable, when following definition was met: (i) absence of detecting differences between the conditions in the applied statistical test (rejection of the alternative hypothesis), and (ii) the presence of a significant positive correlation with an at least moderate effect size between the assessed conditions. Secondly, we tested for differences in effectiveness between SICI and ICF within every condition. Thirdly, the MEP amplitudes of SICI and ICF were compared with the test-pulse amplitude to see, if there was effective modulation in the dpTMS protocols. Lastly, we tested for differences of TS amplitude between conditions. All values in text, figures, and tables are depicted as mean ± SEM.

## Results

### Condition Comparisons

We tested whether there was a difference in the MEP amplitude between the four different conditions for the different ISIs (SICI and ICF). Analysis of SICI 1 showed a significant condition effect χ^2^ (2) = 7.00, *p* = 0.030. However, *post hoc* pairwise comparisons did not show any significant differences. There was a significant difference between conditions for SICI 3 χ^2^ (3) = 20.14, *p* < 0.001. *Post hoc* analysis showed that MS PA (56.78 ± 12.94 %) was significantly larger than MS AP (9.52 ± 1.70 %, *p* = 0.005). MS AP was significantly smaller than MV AP-PA (49.17 ± 11.11 %, *p* = 0.002) and smaller than MV PA (42.05 ± 10.84 %, *p* = 0.007). There were no overall significant differences between conditions for ICF 10 χ^2^ (3) = 6.43, *p* = 0.093 or between conditions for ICF 15 χ^2^ (3) = 0.94, *p* = 0.815. To further assess for potential underlying associations between conditions, we calculated Spearman’s rank-order correlations. The analysis of the conditions in SICI 1 showed no significant correlations. In the SICI 3, all the conditions were tested except for the MS AP condition. There was a significant correlation between the MS PA and the MV AP-PA condition *r_s_* = 0.657, *p* = 0.039. MS PA highly correlated with MV PA *r_s_* = 0.829, *p* = 0.001. Finally, there was a trend between MV AP-PA and MV PA *r_s_* = 0.600, *p* = 0.078. There were no significant correlations between the ICF 10 conditions as well as the ICF 15 conditions.

**FIGURE 2:**
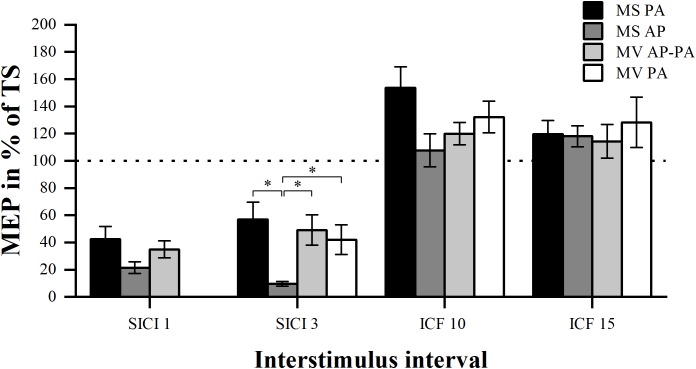
Modulation compared per condition. Comparison of MEP modulation induced by the four different stimulator current direction conditions sorted by ISI (1, 3, 10, and 15 ms). ^∗^*p* < 0.05, bonferroni-corrected.

In summary, the MS PA condition was classified comparable to the MV AP-PA and the MV PA conditions within the framework of our definition, when assessing SICI at an 3 ms ISI, for details please see Figure 2 and Table 2.

**Table 2:** Association between conditions.

Condition	SICI 1		SICI 3		ICF 10		ICF 15	
	Rho	*p-value*	Rho	*p-value*	Rho	*p-value*	Rho	*p-value*
MS PA – MS AP	0.165	1.000	n/a	n/a	0.415	0.846	0.297	1.000
MS PA – MV AP-PA	0.407	0.450	0.657	0.039^∗^	0.218	1.000	0.429	0.768
MS PA – MV PA	n/a	n/a	0.829	0.001^∗^	0.178	1.000	0.226	1.000
MS AP – MV AP-PA	-0.095	1.000	n/a	n/a	0.341	1.000	-0.055	1.000
MS AP – MV PA	n/a	n/a	n/a	n/a	0.538	0.300	0.503	0.414
MV AP-PA – MV PA	n/a	n/a	0.600	0.078	0.525	0.342	0.121	1.000

*Spearman’s rank-order correlations between the assessed conditions (MS PA, MS AP, MV AP-PA, and MV PA). ^∗^p < 0.05, bonferroni-corrected.*

### Effectiveness of Stimulation Paradigms

**FIGURE 3:**
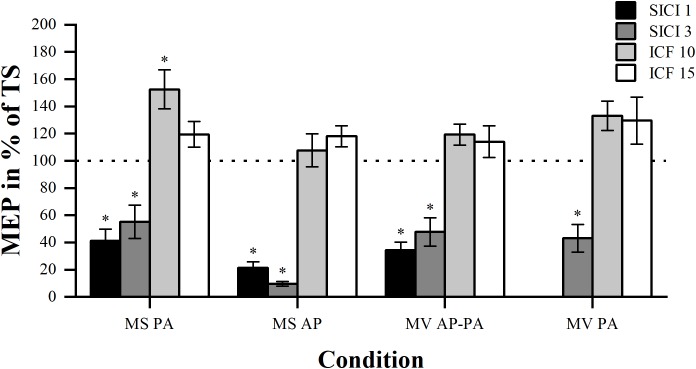
Modulation compared per ISI. Comparison of MEP modulation induced by SICI and ICF at the four different ISIs (1, 3, 10, and 15 ms) sorted by condition. Asterisk indicates significant modulation compared with TS alone. ^∗^*p* < 0.05, bonferroni-corrected.

For every condition, we tested whether the four different ISIs resulted in differential MEP modulation. Furthermore, we compared the two inhibition and facilitation paradigms within the conditions to see whether one of the two ISIs was more effective, please see Figure 3.

As expected, in all conditions the comparison between the four different paradigms showed significant overall differences in MEP modulation. Results are for MS PA: χ^2^ (3) = 30.44, *p* < 0.001, MS AP: χ^2^ (3) = 34.71, *p* < 0.001, MV AP-PA: χ^2^ (3) = 31.24, *p* < 0.001 and MV PA: χ^2^ (2) = 19.60, *p* < 0.001, respectively. *Post hoc* comparisons showed that there was no significant difference in MEP magnitude between the two inhibitory paradigms in all the conditions. *Post hoc* comparisons within the facilitation paradigms did also show that there were no significant differences. Overall, different ISIs did not result in significantly different MEP modulation within the inhibitory or facilitatory paradigms.

### Modulation Effect

We tested whether the MEP amplitudes assessed at different ISIs were significantly different from the TS, to show whether modulation was present. The results showed that all the inhibitory paradigms resulted in significant modulation for all conditions, please see Table 3. However, for the facilitatory paradigms only ICF 10 in MS PA resulted in a significant modulation.

**Table 3:** Modulation effect.

Condition	SICI 1	SICI 3	ICF 10	ICF 15
	*p*-value	*p*-value	*p*-value	*p*-value
MS PA	0.003^∗^	0.034^∗^	0.034^∗^	1.000
MS AP	0.001^∗^	0.001^∗^	1.000	0.245
MV AP-PA	<0.001^∗^	0.012^∗^	0.302	1.000
MV PA	n/a	0.003^∗^	0.288	0.332

*Modulation effect for every ISI compared with the TS. Significant results depict that there was significant inhibition or facilitation compared with the TS. ^∗^p < 0.05, bonferroni-corrected.*

### Auxiliary Analysis

**FIGURE 4:**
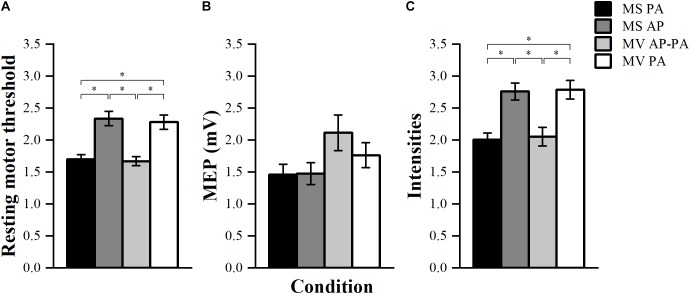
Resting motor threshold, TS amplitude, and stimulator intensities. **(A)** Normalized resting motor threshold (RMT) across conditions. **(B)** Achieved peak-to-peak amplitude for the TS across different conditions. **(C)** Normalized stimulator output intensity at the achieved TS amplitude (∼1 mV) across conditions. ^∗^*p* < 0.05, bonferroni-corrected.

To compare the RMT between stimulators, the threshold was normalized to the maximal energy stored in the stimulator (Kammer et al., 2001). There was a significant effect of condition *F* (3, 39) = 79.01, *p* < 0.001. *Post hoc* comparisons showed that MS PA (1.70 ± 0.07) was significantly smaller than MS AP (2.34 ± 0.11, *p* < 0.001). MS PA was smaller than MV PA (2.28 ± 0.11, *p* < 0.001), MS AP was larger than MV AP-PA (1.67 ± 0.07, *p* < 0.001) and MV AP-PA was smaller than MV PA (*p* < 0.001), see Figure 4A.

The achieved TS amplitudes are depicted in Figure 4B. Results showed that there were significant differences between the four conditions in terms of TS amplitude *F* (3, 39) = 3.02, *p* = 0.041. However, *post hoc* pairwise comparisons did not show any significant differences between the conditions.

The stimulator output intensities to achieve a TS MEP of ∼1 mV were normalized to the maximal energy stored in the stimulator. The four conditions turned out to be significantly different from each other *F* (3, 39) = 77.99, *p* < 0.001. *Post hoc* comparisons showed that MS PA (2.01 ± 0.10) was significantly smaller than MS AP (2.76 ± 0.13, *p* = 0.001), MS PA was smaller than MV PA (2.79 ± 0.14, *p* = 0.001), MS AP was larger than MV AP-PA (2.05 ± 0.15, *p* = 0.003) and MV AP-PA was smaller than MV PA (*p* = 0.002), see Figure 4C.

## Discussion

In summary, we were not able to detect differences for the assessed conditions (MS PA, MS AP, MV AP-PA, and MV PA) for SICI 1, ICF 10, and 15. For SICI 3 the AP current direction led to stronger inhibition. This current direction dependent effect is discussed below.

Subsequently, we evaluated if the conditions are comparable by performing correlation analysis, which suggests that the MS PA condition is comparable with the MV AP-PA, and MV PA conditions, when assessed at an ISI of 3 ms. This finding enables the combination of SICI 3 data sets, which is often an important prerequisite for efficient meta-analysis or open science approaches. Being able to utilize different TMS systems and waveforms further strengthens the potential of SICI 3 to develop into a biomarker suitable for predicting motor recovery (Liuzzi et al., 2014) or treatment response (Zimerman et al., 2012).

### Effect of Current Direction

The current direction dependent effect for SICI demonstrated here has been described previously in the literature with the largest difference at an ISI of 3 ms (Hanajima et al., 2008). A proposed mechanism may be that SICI affects mainly later I-waves (Nakamura et al., 1997) and these are mainly targeted by AP currents. In contrast, PA currents mainly evoke I1-waves (Sakai et al., 1997). Depending on the specific research questions, it might be useful to study SICI with different current directions including the unconventional AP direction. This can provide additional information, e.g., when assessing underlying mechanisms of neurological conditions (Hanajima et al., 2008, 2011). A practical technical note to be mentioned is that when utilizing the Magstim BiStim^2^ stimulator with a standard D70 alpha flat coil the AP technique is manually more demanding, when the equipment does not provide a switch-option to change the current direction within the coil. Furthermore, the AP condition requires higher stimulation intensities for RMT and 1 mV MEP (Kammer et al., 2001), which could limit its application in specific conditions with increased thresholds, such as in healthy aged populations (Sale et al., 2016) or within neurological disorders such as stroke (McDonnell and Stinear, 2017).

### Effect of Interstimulus Interval

For SICI two different phases, an early at ∼1 ms and a late at ∼2.5 ms, have been reported (Fisher et al., 2002). These phases seem to have different thresholds and a differential susceptibility toward voluntary muscle activation. Furthermore, they show a low correlation and are most likely mediated by different inhibitory circuits (Roshan et al., 2003). For a comparable CS intensity as used in our study (80% of RMT) a monophasic waveform with a PA current direction has resulted in comparable levels of inhibition for both phases (Roshan et al., 2003). The present study was able to replicate these previous findings. For the AP current direction, we found a trend for stronger inhibition at an ISI of 3 ms compared with 1 ms. Moreover, we found a trend for more facilitation at the 10 ms ISI compared with the 15 ms for the ICF paradigm in the Magstim PA condition. Both may be explained by different threshold levels of the underlying neuronal circuits.

### Effect of Waveform

To study the effects of waveform is important since it is assumed that the underlying effect is mediated by different activation sites. Main evidence is based on the recording of TMS-induced descending volleys sampled with epidural recordings (Di Lazzaro et al., 2008). In these, monophasic PA currents mainly affect I-waves at lower intensities, which suggest a main activation site at first and higher-order excitatory interneurons. At higher intensities monophasic PA pulses can also induce a small D-wave, resulting from activation of the proximal part of a pyramidal cell axon. In contrast, monophasic AP currents preferentially induce later I-waves, pointing toward a primary activation site at higher-order excitatory interneurons. Biphasic AP-PA currents result in descending volleys at a slightly different latency and periodicity. The fact that both phases can activate neuronal elements suggests a more widespread activation. In addition, the different waveforms may also affect slightly different neuronal populations as the cortical folding and its impact on the axonal orientation results in different susceptibilities of the targeted cortical neurons (Di Lazzaro et al., 2008).

The effects of waveforms on SICI and ICF was recently investigated by Davila-Pérez et al. (2018). They found less inhibition for a biphasic pulse when compared with a monophasic pulse at a 3 ms ISI in their *post hoc* testing, without a significant main effect. We were not able to replicate these results. Small effects size (no significant main effect) and difference in TS adjustment (120% of RMT versus adjusted to ∼1 mV MEP) may have contributed to the differential findings. Furthermore, Davila-Pérez et al. (2018) reported significant less facilitation for ICF with monophasic waveform and a PA current direction, when compared with the biphasic waveform. These findings were not apparent in our current data, though measured at a different ISI (12 ms versus 10 or 15 ms). A possible explanation for the similarity of the induced effects for the monophasic PA and biphasic AP-PA condition, in our data, could be the similar pattern of supposedly recruited descending volleys (Di Lazzaro et al., 2001).

### No Consistent Effect of ICF

It is of note, that we could not find significant facilitation for the ICF paradigm when compared to TS for most conditions, except for the conventional Magstim PA condition at a 10 ms ISI. This complements available literature, which reports low reliability for ICF (Boroojerdi et al., 2000; Maeda et al., 2002; Fleming et al., 2012) and limits its comparability. Discussed underlying biological sources of variability are asynchrony and phase cancelation of descending volleys, inherent changes in cortical excitability (Boroojerdi et al., 2000), and different thresholds for SICI and ICF (Hermsen et al., 2016). Our result for the unconventional AP ICF contradicts the finding of Davila-Pérez, who found only significant facilitation with the AP current direction (Davila-Pérez et al., 2018). A possible reason for the differential findings between these studies might be due to the fact that different ISIs were studied – 10 and 15 ms versus 12 ms.

### Limitations

We have identified a few limitations of our study. Our adjustment of the TS amplitude tended to be larger than the aimed 1 mV peak-to-peak amplitude (MS PA: 1.46 ± 0.16 mV, MS AP: 1.48 ± 0.17 mV, MV AP-PA: 2.11 ± 0.28 mV, and MV PA: 1.76 ± 0.19 mV). However, our amplitudes were well in the comparable range for SICI (Sanger et al., 2001; Garry and Thomson, 2009). The impact for ICF paradigms might be larger, since the effect of ICF seems to decrease at higher TS amplitudes (assessed target amplitude 4 mV) (Sanger et al., 2001). However, the range around 4 mV is much higher than in our study. We cannot exclude that suboptimal TS adjustment may have contributed to the inconstant facilitation we found for ICF. Moreover, the Magstim and MagVenture coils differ in design, e.g., inductance, angle of the surface, overlap of the wire loops, please see Table 1. Though, they share comparable values for focality and stimulation depth (Deng et al., 2013) and seem to trigger similar physiological effects (Thielscher and Kammer, 2004). It is of note, that in the present study specific intensities and the above mentioned coil designs, which are currently mainly used (Rossi et al., 2009; Deng et al., 2013), have been tested and not the full possible range of parameters for paired pulse paradigms. This should be taken into account, when prospective assumptions are made toward a larger stimulation parameter space. For statistical analyses, we *a priori* defined to apply Bonferroni correction, a well-established, but rather conservative correction method. In upcoming studies more liberal statistical approaches can be applied to further support the current findings. Furthermore, a rather small sample size may have restrained us from detecting effects with low effect sizes. Lastly, we did not use a coil tracking system, which may improve coil positioning (Washabaugh and Krishnan, 2016). Although, for motor-cortex centered SICI assessments hand-held and navigated approaches have shown to result in comparable reliability (Fleming et al., 2012).

## Conclusion

In summary, we obtained comparable results for SICI 3, when comparing MS PA to MV AP-PA and MV PA. This opens the opportunity to combine data sets sampled with different experimental setups, supports conduction of multi-center trials, and enables between study comparisons toward open science.

## Data Availability

The datasets generated for this study are available on request to the corresponding author.

## Ethics Statement

All subjects gave written informed consent in accordance with the Declaration of Helsinki (World Medical Association, 2013). The protocol was approved by the cantonal ethics committee Vaud, Switzerland (Project Number 2017-00765).

## Author Contributions

MW and FH developed the study concept and wrote the study protocol. MW and TM implemented the experimental setup. MW and LD collected the data. LD processed and analyzed the data, and performed the statistical analysis. MW, LD, TM, and FH were involved in the data interpretation. MW and LD searched the literature and wrote the first draft of the manuscript. MW, LD, TM, and FH revised and approved the final version of the manuscript.

## Conflict of Interest Statement

The authors declare that the research was conducted in the absence of any commercial or financial relationships that could be construed as a potential conflict of interest.
